# Dynapenia, Dehydroepiandrosterone (DHEA), and Redox Balance in Geriatric Patients—Does Sex Play a Role?

**DOI:** 10.3390/nu17213413

**Published:** 2025-10-30

**Authors:** Jakub Śliwowski, Aleksandra Wojszel, Justyna Rentflejsz, Joanna Rogalska, Małgorzata Michalina Brzóska, Zyta Beata Wojszel

**Affiliations:** 1 Interdisciplinary Students’ Scientific Group, Department of Geriatrics, Medical University of Bialystok, 15-089 Bialystok, Poland; 42216@student.umb.edu.pl (J.Ś.); 42230@student.umb.edu.pl (A.W.); 2 Doctoral School, Medical University of Bialystok, 15-089 Bialystok, Poland; justyna.rentflejsz@sd.umb.edu.pl; 3 Department of Toxicology, Medical University of Bialystok, 15-089 Bialystok, Poland; joanna.rogalska@umb.edu.pl (J.R.); malgorzata.brzoska@umb.edu.pl (M.M.B.); 4 Department of Geriatrics, Medical University of Bialystok, 15-089 Bialystok, Poland

**Keywords:** dynapenia, total oxidative status, total antioxidative status, oxidative stress index, dehydroepiandrosterone, sex differences

## Abstract

Background: Dynapenia is an age-related decline in muscle strength that leads to unfavorable outcomes, decreased functional capacity, and increased mortality. The study aimed to measure total oxidative status (TOS) and total antioxidative status (TAS), and to explore the role of oxidative stress in dynapenia, with consideration of sex differences and dehydroepiandrosterone (DHEA) influence. Methods: The study was performed in geriatric ward patients over 60 years of age, who were able to take part in the functional assessment. Dynapenia was diagnosed if grip strength was <27 kg in men, and <16 kg in women. DHEA, TOS, and TAS were assayed in the serum. The severity of oxidative stress was expressed as the oxidative stress index (OSI). One hundred and thirty-four patients (73.9% women, mean age 79.1 ± 7.3 years) took part in the study. Results: Dynapenia was observed in 37.3% of cases, with similar prevalence in women (35.4%) and men (42.9%). The logistic regression analysis identified older age, higher OSI, lower BMI, and lower DHEA as significant determinants of dynapenia, while sex was not a significant factor. Conclusions: The study demonstrates a significant association between oxidative stress and dynapenia in older adults, independent of sex and comorbidity burden. The observed sex-specific patterns—especially the stronger association in women—and the link between lower DHEA levels and dynapenia underscore the importance of hormonal regulation in this process.

## 1. Introduction

Oxidative balance, representing the homeostasis between pro-oxidant and antioxidant mechanisms, plays a critical role in maintaining cellular integrity and systemic physiological function [1,2]. Redox balance can be assessed by the ratio of total oxidative status (TOS) to total antioxidative status (TAS), expressed as the oxidative stress index (OSI) [3]. Oxidative stress arises from an excess of reactive oxygen species (ROS) relative to antioxidant defenses, disrupting homeostasis and activating pro-inflammatory pathways [1]. There are marked sex-related differences in oxidative and inflammatory stress mechanisms, which evolve with aging. Studies indicate that men generally exhibit higher basal inflammation, greater ROS production, and less efficient antioxidant defenses, which may partly explain their shorter life expectancy [4]. These differences tend to diminish with age due to the loss of estrogen’s antioxidant effects in postmenopausal women, accelerating aging processes and attenuating sex differences in oxidative stress markers [4,5]. Nevertheless, the longer lifespan of women remains evident. This may be partly attributable to the slower accumulation of oxidative damage across the lifespan compared to men, although additional factors likely contribute. For instance, significant sex differences are observed in the prevalence of numerous diseases, including cardiovascular disorders such as hypertension, heart failure, myocardial infarction, cardiac hypertrophy, and shock—conditions in which oxidative stress plays a well-established pathogenic role [6].

One of the unfavorable prognostic changes observed with aging is dynapenia, defined as an age-related decline in muscle strength independent of underlying neuromuscular or muscular pathology [7]. Although it may occur across all age groups, it is most prevalent among older adults. Dynapenia is associated with increased risk of falls, reduced functional capacity, impaired psychological well-being, and higher mortality [8,9,10,11]. Multiple processes underlie the pathogenesis of sarcopenia and dynapenia in older adults. Inflammatory mechanisms—including low-grade chronic inflammation, often referred to as “inflammaging”—and oxidative stress are considered key contributors [12,13]. The interplay between chronic inflammation and oxidative stress is thought to accelerate the progressive decline in muscle strength observed in dynapenia through several mechanisms operating at different sites within the muscle fiber. This results in a deterioration of skeletal muscle function with advancing age, caused by both a reduction in muscle mass due to a negative protein balance (quantitative changes) and a decrease in intrinsic force-generating capacity due to impairments in neuromuscular junction activation, excitation–contraction coupling at the ryanodine receptor (RyR), and cross-bridge cycling within the myofibrillar apparatus (qualitative changes) [14].

Hormonal imbalances can contribute to the development of sarcopenia and negatively affect muscle strength in the general population. Several hormones have been implicated, including growth hormone, insulin, thyroid hormones, vitamin D, and sex hormones (testosterone and estrogens), all of which decline with age [15,16,17]. Dehydroepiandrosterone (DHEA) and dehydroepiandrosterone sulfate (DHEAS) are adrenal hormones that undergo peripheral conversion into physiologically active testosterone and estradiol. The age-related decline in DHEA/DHEAS levels—referred to as adrenopause) [18]—may also play a significant role, while the available findings remain scarce and inconsistent.

A better understanding of the interrelationships between oxidative stress, dynapenia, and sex-related determinants could substantially improve strategies for the prevention and management of sarcopenia, ultimately contributing to increased longevity and improved quality of life in old age. Therefore, the present study aimed to measure TOS and TAS levels and evaluate the degree of oxidative stress in the serum of older patients with low muscle strength, exploring the role of oxidative stress in dynapenia. Furthermore, the study examined relationships between these parameters and patient sex, taking into account the influence of dehydroepiandrosterone.

## 2. Materials and Methods

### 2.1. Study Participants

The study was performed in geriatric ward patients over 60 years of age, who gave their informed consent to participate and were able to take part in the functional assessment. Patients with Parkinson’s disease, moderate to severe dementia (Mini-Mental State Examination < 18 points), severe chronic obstructive pulmonary disease (COPD) on oxygen therapy, severe osteoarthritis, a pacemaker, and on active anti-neoplasm treatment were excluded from the study. The study was approved by the Bioethics Committee at the Medical University of Bialystok (no. APK.002.513.2022) on 15 December 2022. All procedures performed in the study complied with the Helsinki Declaration and its subsequent amendments.

We collected information on patient’s age, sex, physical activity (asking about walking, carrying out chores, mowing the lawn, gardening, hiking, jogging, cycling, dancing, aerobics, swimming—based on the short version of the Minnesota Leisure Time Activity questionnaire; for each activity, information also was obtained on the frequency and duration of participation in the activity which was used to estimate the kilocalories of energy expended per week in physical activity) [19], and prevalence of chronic diseases, the pathogenesis of which involves oxidative stress and chronic inflammation (hypertension, orthostatic hypotension, atrial fibrillation (AF), chronic heart failure (CHF), ischemic heart disease (IHD), peripheral arterial disease (PAD), chronic obstructive pulmonary disease (COPD), chronic kidney disease (CKD), chronic arthritis, dementia, diabetes, and history of neoplasm, myocardial infarction, and stroke).

### 2.2. Functional Assessment and Nutritional Health

Functional assessment was a part of the routine comprehensive geriatric assessment conducted in the department, and included the following:

- Measurement of hand grip strength of the dominant hand with a manual hydraulic dynamometer SAEHAN DHD-1 (Glanford Electronics Ltd., Scunthorpe, UK) (mean of two results);
- The assessment of the risk of falls with the Timed Up and Go test (TUG) [20];
- Gait speed measurement evaluation during the 4.57 m walk at usual pace;
- Screening for sarcopenia with the SARC-F questionnaire (a self-assessment of deficiencies in strength, the need for assistance with walking, problems while rising from a chair and climbing stairs, and experiencing falls) [21,22];
- The ability to perform activities of daily living (ADL) with the Barthel Index [23];
- The ability to perform instrumental activities of daily living (IADL) with the 6 instrumental ADL items of Duke OARS scale [24];
- Emotional health assessment with the 15-item Geriatric Depression Scale (GDS) [25];
- Cognitive abilities assessment with the Short-Blessed Scale [26].

Nutritional health was evaluated with body mass index (BMI), waist circumference (WC), and the Mini Nutritional Assessment-Short Form (MNA-SF) [27].

### 2.3. Laboratory Data and Biochemical Parameters

Data on serum albumin, protein, 25-hydroxycholecalciferol (25 (OH) D), creatinine (SCr), N-terminal pro-B-type natriuretic peptide (NT-proBNP), thyroid-stimulating hormone (TSH), and C-reactive protein (CRP), total lymphocytes count, and hemoglobin (Hb) were collected if they were determined within the patient’s hospital stay. Fasting venous blood was used to measure their serum concentrations.

Serum samples for TOS, TAS, Interleukin-6 (IL-6), tumor necrosis factor alpha (TNF-α), and dehydroepiandrosterone (DHEA) measurements were obtained from fasting venous blood and then stored at −80 °C until assayed. Blood samples were collected during scheduled blood draws.

The concentrations of TNF-α, IL-6, and DHEA in the serum were measured using commercial kits that employ a quantitative double-antibody sandwich enzyme-linked immunosorbent assay (sandwich ELISA). The methodology is based on the characteristics of a target analyte (the parameter being measured) that contains epitopes of the antigen, which can simultaneously be recognized by both the pre-coated capture antibody and the detection antibody. These antibodies identify the epitopes of the antigen, which are situated (“sandwiched”) between the capture antibody and the detection antibody. Initially, the capture antibody is immobilized on a plate, and then the analyzed sample containing the antigen is added. Next, the ELISA detection antibody binds to the antigen in proportion to the quantity of the target analyte, forming this “sandwich”. The activity of the linked enzyme is then measured after adding the substrate, and the resulting signal is detected at 540 nm using a spectrophotometer. All measurements were conducted according to the manufacturer’s instructions provided in the kits and were performed in duplicate, utilizing two kits for each parameter. An Epoch spectrophotometer (BioTek Instruments, Inc., Winooski, VT, USA) was used.

The concentration of TNF-α was quantified utilizing the Human TNF-α Quantikine™ ELISA Kit (No. DTA00D) from Bio-techne R&D Systems, Inc. (Abingdon, UK). The intra-assay CV was <2.2% for the first kit and <3.2% for the second kit; the inter-assay CV was <4%. The IL-6 concentration was assayed employing the Human IL-6 ELISA Kit (No. EH0201) provided by FineTest (Wuhan, China) with the intra-assay CV < 10% (<10% for the first kit and <4.3% for the second kit) and the inter-assay CV < 2%. The concentration of DHEA was measured in duplicate using the Human Dehydroepiandrosterone (DHEA) ELISA Kit (No. MBS266093) from MyBioSource (San Diego, CA, USA), based on a sandwich ELISA. The assay was conducted according to the manufacturer’s instructions. The intra-assay CV was <3% for the first kit used and <2% for the second kit; the inter-assay CV was <2%.

TOS and TAS were measured in the serum using spectrophotometric PerOx (TOS/TOC) Kit (No. KC5100) and ImAnOx (TAS/TAC) Kit (No. KC5200), respectively, provided by Immundiagnostik AG (Bensheim, Germany). To assay TOS, total lipid peroxides present in the serum were quantified through the reaction of peroxidase and peroxides present in the tested sample and the transformation of 3,3′,5,5′-tetramethylbenzidine added to the sample into a colored product. TOS values measured in control samples included with the first kit and the second kit fell within the ranges specified by the manufacturer, and the intra-assay coefficient of variation (CV) was <2% for the first kit and <4% for the second kit. TAS was determined based on the reaction of antioxidants present in the serum and the added hydrogen peroxide. TAS values determined in control samples included with the first kit and the second kit fell within the ranges specified by the manufacturer. The intra-assay CV was <1% for the first kit and <2% for the second kit, while the inter-assay CV was <2%.

### 2.4. Study Parameters

Dynapenia was diagnosed if grip strength was <27 kg in men and <16 kg in women. Cut-off points for low performance were established according to EWGSOP2 consensus for sarcopenia diagnosis and included slow gait speed (equal or lower than 0.8 m/s) and TUG equal or higher than 20 s [28]. The result of SARC-F ≥ 4 points indicated probable sarcopenia and a potential risk for poor functional outcomes [21,22]. Obesity was diagnosed if BMI ≥ 30 kg/m^2^ and malnutrition if BMI < 18.5 kg/m^2^. Abdominal obesity was diagnosed if WC ≥ 94 cm in men and ≥80 cm in women. Low physical activity was defined according to Fried et al.’s criteria used to define frailty—approximately <383 Kcal of physical activity per week for men and <270 Kcal for women (based on self-reported physical activity) [29]. The co-occurrence of two or more of the assessed chronic diseases was treated as multimorbidity. Patients who had <9 points in the Duke OARS IADL scale were treated as IADL-dependent, and those who collected <80 points in the Barthel Index were treated as ADL-dependent. A result >4 points in the GDS pointed to probable depression and >9 points in the Short-Blessed scale—to cognitive impairment. Patients who had <8 points in the MNA-SF scale could be diagnosed as malnourished, and having 8–11 points—as at risk of malnutrition. Hypoproteinemia was diagnosed if total serum protein was below 6 g/dL and hypoalbuminemia if serum albumin was below 3.5 g/dL. Anemia was defined as Hb levels <13.0 g/dL in men and <12.0 g/dL in women.

OSI was mathematically calculated as the ratio of TOS and TAS (OSI = TOS/TAS) [30]. GFR was calculated with the Cocroft–Goult equation:

GFR C − G (mL/min) = (140 − age in years) × (weight in Kg) × 1.23 if male (1.04 if female)/SCr in μmol/L).

### 2.5. Statistical Analysis

Statistical analysis was performed with IBM SPSS Version 18 Software suite (SPSS, Chicago, IL, USA). Variables’ distribution was checked with the Shapiro–Wilk test and presented as frequencies and percentages (categorical variables), as means and standard deviations (normally distributed quantitative variables), or as medians and interquartile ranges (not normally distributed quantitative variables). Proportions were compared using χ^2^ tests, while Student’s *t*-test for independent samples and the Mann–Whitney U test were used to compare means and medians. Odds ratios with 95% confidence interval (CI) and *p* value were estimated by multivariable logistic regression methods (direct and stepwise backward) with dynapenia as outcome variable. Dynapenia predictors with a *p* value ≤ 0.1, without those highly correlated to avoid multicollinearity effect, were included. In all analyses, a two-tailed *p* value of less than 0.05 was regarded as significant. Analyses did not include missing data.

## 3. Results

### 3.1. Study Group Characteristic

One hundred and thirty-four patients (73.9% of women; mean age 79.1 ± 7.3 years) participated in the study. Table 1 presents the study group characteristics. The women and men studied did not differ significantly in terms of age, physical activity, nutritional status (they obtained similar scores on the MNA-SF, and a similar percentage of individuals with MNA_SF < 8 points), or functional status. Women were more likely to have GDS scores that could indicate depression, assessed their functional capacity in instrumental ADLs as lower, and had slightly worse scores on the Barthel scale. However, overall, the participants were quite competent in self-care. Their cognitive function was similar on the Short-Blessed Katzman Test. Sarcopenia was twice as likely to be suspected in the women’s group based on the SARC-F test compared to men, but a slightly higher percentage of male patients met the criterion for dynapenia, although this difference was not statistically significant.

### 3.2. Characteristics of Participants with Low and Normal Hand Grip Strength

Characteristics of participants with and without dynapenia are presented in Table 2. The “dynapenia+” group and patients with proper HGS did not differ in sex, in percentage of people with hypertension, atrial fibrillation, ischemic heart disease, periphery artery disease, history of brain infarct, myocardial infarction orthostatic hypotension, COPD, asthma, diabetes, chronic kidney disease, osteoarthritis, dementia, depression, or neoplasm. They differed significantly in the mean age (82.3 ± 6.3 versus 77.3 ± 7.3), the percentage of people with heart failure (56% versus 34.5%, *p* = 0.02), the median number of chronic diseases (5; IQR, 4–6 versus 4; IQR, 3–5 in the “dynapenia−” group, *p* = 0.005), and the percentage of people with multimorbidity (100% versus 91.7%, *p* = 0.045). People with dynapenia were significantly more frequently dependent in ADL and instrumental ADL, had higher scores in Barthel Index and Duke OARS IADL, and significantly more frequently had >9 points in Short-Blessed scale. Low HGS was significantly less frequently observed in patients with higher BMI and, less frequently, BMI > 30 kg/m^2^ was observed in patients with dynapenia. Patients with dynapenia more frequently fulfilled the latest European consensus criteria for sarcopenia and severe sarcopenia—SARCF ≥ 4 points, slow gait and TUG ≥ 20 s. They had more frequently total serum protein < 6 g/dL, significantly lower mean serum albumin, median GFR, median concentration of DHEA, and significantly higher NT-proBNP concentration. They did not differ in several characteristics analyzed, including inflammatory parameters (CRP, IL-6, and TNF-α).

### 3.3. Dynapenia and Redox Status

There was no difference observed between the “dynapenia+” and “dynapenia−” groups within TAS and TOS measurements, but the difference in the case of OSI was statistically significant (Table 3). The analysis revealed that redox balance status was associated with dynapenia only among women. In this case, both TOS and OSI were significantly higher (by 25.4% and 31%, respectively) in dynapenic women than in women with normal HGS. In contrast, there was no significant difference in TAS. In men, no difference in TOS, TAS, and OSI was observed between “dynapenia+” and “dynapenia−” cases.

### 3.4. Determinants of Low HGS in Logistic Regression Analysis

A direct multivariable logistic regression analysis was carried out on dynapenia (low HGS) as outcome and 17 predictors: age, heart failure, history of myocardial infarction, neoplasm, number of chronic diseases, MNA-SF, BMI, serum protein < 6/dL, hemoglobin, NT-proBNP, GFR, DHEA, OSI, serum albumin, low physical activity, GDS, and Short-Blessed test (Table 4). A few variables meeting the criterion *p* ≤ 0.1 (multimorbidity, BMI ≥ 30 kg/m^2^, GFR < 60 mL/min/1.73 m^2^) were not included in the logistic regression after testing for correlation with other variables and for their multicollinearity effect. Significantly higher odds for low HGS were observed for age (odds ratio, 1.13; 95% CI, 1.02–1.26; *p* = 0.04), history of myocardial infarction (odds ratio, 19.01; 95% CI, 1.20–302.25; *p* = 0.03), and OSI (odds ratio, 1.87; 95% CI, 1.05–3.34; *p* = 0.04). There was also a trend for an association with variable “BMI” (odds ratio, 0.90; 95% CI, 0.80–1.01; *p* = 0.06), and DHEA (odds ratio, 0.09; 95% CI, 0.01–1.01; *p* = 0.05) when controlling for heart failure, neoplasm, number of chronic diseases, MNA-SF, serum protein < 6/dL, hemoglobin, NT-proBNP, GFR, serum albumin, low physical activity, GDS, and Short-Blessed test (Model 1). An overall prediction success rate of 80.2% was observed, with 74.4% of the “dynapenia+” status (sensitivity) and 84.1% of “dynapenia−” status (specificity) correctly predicted (Nagelkerke’s R-square = 0.475). After adjusting for the variable “sex” (as biologically important covariate, despite its not significant association with dynapenia in bivariate analysis), the odds connected with “history of MI” became a not significant one (odds ratio, 18.42; 95% CI, 1.03–329.80; *p* = 0.05), whereas DHEA become a significant one (odds ratio, 0.08; 95% CI, 0.01–0.88; *p* = 0.04)—Model 2. For the second model, an overall prediction success rate of 82.1% was observed, with 76.7% of the “dynapenia+” status (sensitivity) and 85.7% of “dynapenia−” status (specificity) correctly predicted (Nagelkerke’s R-square = 0.483).

A stepwise backward logistic regression analysis was performed on dynapenia as outcome and 18 predictors constructing Model 2 (Table 5). The results of the analysis suggested the model with 7 variables only: age (odds ratio, 1.14; 95% CI, 1.05–1.24; *p* = 0.002), BMI (odds ratio, 0.89; 95% CI, 0.82–0.98; *p* = 0.01), OSI (odds ratio, 1.78; 95% CI, 1.08–2.95; *p* = 0.02), DHEA (odds ratio, 0.11; 95% CI, 0.02–0.88; *p* = 0.04), and three variables with a trend of association—MI, CHF, and neoplasm. Prediction success was 80.2% in the case of Model 3, with 72.1% of “dynapenia+” cases and 85.7% of “dynapenia–“ cases correctly predicted (Nagelkerke’s R-square = 0.437)**.**

## 4. Discussion

Muscle strength assessment has become an essential component of health and fitness evaluation in geriatric patients. All expert groups involved in studies on sarcopenia, its definition, and the development of diagnostic criteria agree that it represents a fundamental parameter that should be measured and considered in this process. The latest European consensus on sarcopenia (published by EWGSOP2) prioritized muscle strength as a key prognostic factor and proposed dynapenia (low muscle strength, e.g., grip strength < 27 kg in men, and <16 kg in women) as the primary screening criterion [28]. Likewise, the position statement of the American Sarcopenia Definition and Outcomes Consortium confirmed that muscle weakness—being a predictor of adverse health-related outcomes such as mobility limitation, falls, disability in activities of daily living (ADL), and mortality among community-dwelling older adults—should be included in the definition of sarcopenia and defined using grip strength with sex-specific cut points [31]. The first global consensus on the conceptual definition of sarcopenia, published in 2024, established the foundation for future harmonized operational criteria, recommending that muscle mass, muscle strength, and muscle-specific force be incorporated into the diagnostic algorithm, while physical performance should be regarded as an outcome measure [32].

The prevalence of dynapenia in the study group, assessed based on handgrip strength (HGS) and according to the EWGSOP2 criteria, did not differ significantly between men and women. However, as expected, the absolute HGS values were significantly higher in men. In population-based studies, dynapenia has been shown to be more prevalent among community-dwelling women than men, and similar trends are observed for sarcopenia itself [33]. The prevalence of dynapenia increases with age, and muscle strength declines more rapidly than muscle mass, suggesting that factors beyond lean body mass contribute to changes in muscle strength [7,34,35]. In our study, dynapenia was also more frequently observed with advancing age, and older age was identified as a significant independent determinant of dynapenia in the logistic regression model, together with lower BMI, higher OSI, and lower DHEA levels. The model was adjusted for heart failure, history of myocardial infarction and neoplasm, number of chronic diseases, MNA-SF score, serum protein concentration (<6/dL), hemoglobin level, NT-proBNP, GFR, serum albumin, low physical activity, GDS-15 score, Short-Blessed test score, and sex.

In randomly selected population-based groups, unlike in our study—which included patients admitted to a geriatric ward with a higher degree of morbidity and various disabilities—men and women generally differ in multiple characteristics related to health status and functional capacity. In contrast, in our study, men and women did not differ in several such factors. Statistically significant differences were observed only in the risk of sarcopenia assessed using the SARC-F questionnaire (higher in women), Barthel Index scores (higher in women, although the prevalence of ADL disability defined as <80 points was similar between sexes), and waist circumference (significantly higher in men; however, the criterion for abdominal obesity based on this parameter was met by a similar proportion of women and men). Regarding the prevalence of chronic diseases assessed in the study, peripheral artery disease (PAD), previous stroke, and orthostatic hypotension were significantly more common in men, whereas osteoporosis was more prevalent in women.

In our study, women did not differ from men in serum total protein, albumin, TSH and NTproBNP, and DHEA concentrations. However, they had significantly lower levels of hemoglobin (although no differences were observed in the prevalence of anemia), GFR, serum creatinine, and 25 (OH)D. No significant differences between sexes were observed in inflammatory parameters (CRP, IL-6, and TNF-α).

Both sex groups demonstrated similar redox balance parameters, as no significant differences were observed between men and women in TOS, TAS, or OSI values. This finding may suggest that sex-related differences in oxidative balance diminish with age [4,5]. Experimental and clinical data indicate, before menopause, women having an advantage over men not only in terms of lower reactive oxygen species (ROS) production but also in greater antioxidant capacity, which is closely associated with estrogen. This advantage, however, disappears after menopause [6,36]. Nevertheless, evidence from hormone replacement therapy studies suggests that estrogen may be as harmful as it is beneficial, particularly from a cardiovascular perspective. Thus, its overall effect remains unclear, although it may explain some of the observed sex-related differences in cardiovascular diseases [6,37].

The study confirmed a strong association between dynapenia, as assessed by handgrip strength (HGS), and redox imbalance in the studied group. OSI values were significantly higher in participants with dynapenia (2.52 ± 1.2 vs. 2.06 ± 0.9 in non-dynapenic individuals), supporting the significant role of oxidative stress in the pathogenesis of age-related muscle strength decline. However, this association was significant only among women. In this group, not only TOS (reflecting the overall oxidative state of the body) but also OSI (representing the imbalance between oxidative and reductive processes and considered the most precise indicator of oxidative stress [30,38]) were significantly higher in participants with dynapenia. Such a relationship was not observed in men, which may suggest the presence of additional factors modulating the association between dynapenia and redox balance, depending on sex. For instance, the Oxidative Balance Score (OBS)—a comprehensive measure of oxidative balance based on dietary and lifestyle factors—was found to be significantly and negatively associated with sarcopenia in the American National Health and Nutrition Examination Survey (NHANES) 2011–2018, but only among women [39]. These findings support the rationale for adhering to an antioxidant-rich diet, particularly among women, to reduce the risk of sarcopenia.

A study conducted on animal models demonstrated that aging had a more pronounced effect on the skeletal muscle mitochondrial proteome in females, resulting in an increased susceptibility to oxidative posttranslational modifications. The authors suggested that this may be attributed, among other factors, to the age-related decline in 17beta-estradiol signaling in females and the relatively stable testosterone levels observed in males [40]. Nevertheless, the specific mechanisms underlying the development of sarcopenia and dynapenia in both remain incompletely understood, and further research exploring sex-specific determinants is warranted.

No association was found with TAS, suggesting that antioxidant mechanisms are not sufficiently effective, and that the redox balance shifts toward oxidation in individuals with dynapenia. However, logistic regression analysis identified OSI as a significant determinant of dynapenia, whereas sex was not a significant factor. This may indicate that oxidative stress can be considered a pathophysiological mechanism involved in the development of dynapenia in older adults, independent of sex. Skeletal muscles are characterized by a high metabolic rate and are therefore particularly susceptible to damage and dysfunction associated with oxidative stress [13,41,42]. Several studies have also identified oxidative stress as a critical determinant of sarcopenia. This may occur through multiple mechanisms: oxidative stress negatively affects muscle mitochondrial function and the neuromuscular junction, promotes denervation, and leads to atrophy—ultimately impairing muscle contractile function [13,43,44].

DHEA was found to be an independent significant predictor of dynapenia in the logistic regression model. In InCHIANTI study, DHEAS was an independent correlate of muscle strength and calf muscle area in older men [45]. Age-related decline in DHEA/DHEAS is proposed to contribute to sarcopenia and dynapenia by weakening anabolic support and reducing the defense against chronic inflammation and muscle catabolism [46]. DHEA acts as a crucial precursor to sex steroids, including testosterone (a potent androgen) and estrogens. These hormones are vital for muscle anabolism and maintaining muscle mass and strength. The age-related drop in DHEA reduces the pool for local (intracrine) and circulating sex steroid productions, potentially leading to a deficiency in anabolic signals necessary for muscle maintenance. DHEA may also support anabolism by potentially increasing IGF-1 (Insulin-like Growth Factor-1). DHEA/DHEAS exhibit anti-inflammatory and immune-modulating effects. Chronic low-grade inflammation (elevated IL-6, TNF-α, CRP) is associated with aging and muscle catabolism. DHEAS may counteract this by activating PPARα, which antagonizes pro-inflammatory pathways (e.g., NF-κB), thereby reducing muscle catabolism. DHEAS is also noted as potential functional antagonist to glucocorticoids (cortisol), which are muscle-wasting (catabolic) hormones often chronically elevated with age [47].

The final regression model, which included several variables correlated with dynapenia, identified BMI as an independent predictor, with higher BMI associated with lower odds for dynapenia. This finding may indicate a potential protective role of higher BMI in the development of sarcopenia among our patients. On the one hand, existing research suggests that obesity may increase the risk of dynapenia and sarcopenia—indeed, the “sarcopenic obesity” phenotype has been described [48], characterized by higher cardiometabolic risk and poorer outcomes [49]. On the other hand, some studies have reported a protective effect of higher BMI on long-term prognosis [50] and the development of dynapenia [45]. Adipose tissue is the primary site of aromatase activity, contributing to increased peripheral conversion of androgens into estrone (E1) during the postmenopausal period [51].

The final model for dynapenia determination also included previous myocardial infarction, heart failure, and cancer, all of which demonstrated a trend toward a significant odds ratio. Previous studies have shown associations between dynapenia and these, as well as other chronic conditions. However, it is worth emphasizing that our study was designed to exclude participants with Parkinson’s disease, moderate-to-severe dementia, severe chronic obstructive pulmonary disease (COPD), those receiving oxygen therapy, individuals with severe osteoarthritis, pacemaker carriers, and those undergoing active antineoplastic therapy. These exclusion criteria may have influenced the final results.

Nevertheless, several limitations of this study warrant consideration. The study population consisted of hospitalized geriatric patients with a high burden of multimorbidity; thus, the results may not be directly generalizable to community-dwelling older adults. The findings may be generalizable only to populations with similar characteristics—advanced age and a high burden of multimorbidity—and not to the general older adult population. The specific characteristics of our study group, being relatively older and with a higher prevalence of chronic diseases than in most previous studies, may have influenced the observed results, including the finding that redox imbalance was a significant, independent predictor of dynapenia [10]. The cross-sectional design precludes causal inference between oxidative stress and dynapenia. Moreover, while TOS, TAS, and OSI provide valuable information on overall oxidative stress, they are systemic rather than tissue-specific indicators. Although these parameters are useful for exploring the relationship between oxidative stress and the decline in muscle strength, they do not specifically reflect processes occurring within skeletal muscle tissue. Simultaneous assessment of more specific biomarkers of muscle damage or aging—such as muscle-specific enzymes (e.g., creatine kinase, lactate dehydrogenase) or oxidative stress-modified muscle proteins—would enhance the tissue specificity and interpretive strength of the findings. Additionally, the absence of direct measurements of sex hormones, particularly estradiol and testosterone, limits the ability to fully elucidate the hormonal modulation of redox balance and muscle strength.

## 5. Conclusions

In conclusion, our study demonstrates a significant association between oxidative stress and dynapenia in older adults, independent of sex and comorbidity burden. The results suggest that systemic redox imbalance, as reflected by elevated OSI, plays a central role in the decline in muscle strength with aging. Moreover, the observed sex-specific patterns—especially the stronger association in women—and the link between lower DHEA levels and dynapenia underscore the importance of hormonal regulation in this process. Understanding these interrelationships may pave the way for novel preventive and therapeutic strategies targeting oxidative stress and hormonal modulation to preserve muscle function and improve the quality of life in the older population.

## Figures and Tables

**Table 1:** Study group characteristics—differences between men and women in health and nutritional parameters.

Characteristic	All	Women	Men	*p* ^1^	Missing Data
*n* (%)	134 (100)	99 (73.9)	35 (26.1)		
Age (y), M (SD)	79.1 (7.3)	79.6 (7.3)	77.7 (7.4)	0.20	-
Chronic diseases					
Hypertension, *n* (%)	118 (88.1)	89 (89.9)	29 (82.9)	0.36	
Atrial fibrillation, *n* (%)	27 (20.1)	20 (20.2)	7 (20)	1.00	
CHF, *n* (%)	57 (42.5)	38 (38.4)	19 (54.3)	0.12	
IHD, *n* (%)	25 (18.7)	17 (17.2)	8 (22.9)	0.46	
MI, *n* (%)	5 (3.7)	2 (2)	3 (8.6)	0.11	
PAD, *n* (%)	22 (16.4)	12 (12.1)	10 (28.6)	0.03	
Brain infarct, *n* (%)	8 (6.0)	3 (3.0)	5 (14.3)	0.03	
Orthostatic hypotension, *n* (%)	38 (28.4)	23 (23.2)	15 (42.9)	0.03	
COPD, *n* (%)	6 (4.5)	3 (3.0)	3 (8.6)	0.18	
Asthma, *n* (%)	2 (1.5)	2 (2)	0 (0.0)	1.00	
Diabetes, *n* (%)	75 (56)	56 (56.6)	19 (54.3)	0.85	
CKD, *n* (%)	47 (35.1)	34 (34.3)	13 (37.1)	0.84	
Osteoarthritis, *n* (%)	11 (8.2)	7 (7.1)	4 (11.4)	0.48	
Osteoporosis, *n* (%)	53 (39.6)	49 (49.5)	4 (11.4)	<0.001	
Neoplasm, *n* (%)	11 (8.2)	6 (6.1)	5 (14.3)	0.16	
Dementia, *n* (%)	8 (6.0)	5 (5.1)	3 (8.6)	0.43	
Depression, *n* (%)	90 (67.2)	72 (72.7)	18 (51.4)	0.04	
Number of chronic diseases, Me (IQR)	4 (3,6)	4 (3,5)	4 (3,6)	0.58	
Multimorbidity (≥2 diseases), *n* (%)	127 (94.8)	94 (94.9)	33 (94.3)	1.00	
Functional abilities and physical activity					
Barthel Index, points, Me (IQR)	95 (85, 100)	95 (85, 100)	95 (90, 100)	0.03	
ADL dependence, *n* (%)	22 (16.4)	19 (19.2)	3 (8.6)	0.19	
Duke OARS IADL, points, Me (IQR)	9 (7, 11)	9 (7, 11)	9 (7, 11)	0.89	
IADL dependence, *n* (%)	58 (43.3)	44 (44.4)	14 (40.0)	0.70	
GDS, points, Me (IQR)	5 (3, 7)	5 (3, 8)	4 (2, 5)	0.007	
GDS > 4 points, *n*(%)	69 (51.9)	56 (57.1)	13 (37.1)	0.05	
Short-Blessed, points, Me (IQR)	4 (2, 10)	4 (2, 8)	6 (2, 14)	0.10	
Short-Blessed > 9 points, *n* (%)	35 (26.3)	23 (23.5)	12 (34.3)	0.26	1
Low physical activity, *n* (%)	45 (34.1)	31 (32.0)	14 (40.0)	0.41	2
Nutritional parameters					
MNA-SF, points, Me (IQR)	11.5 (9, 13)	12 (9, 13)	11 (10, 13)	0.77	
MNA-SF < 8 points, *n* (%)	16 (11.9)	12 (12.1)	4 (11.4)	0.96	
8–11 points, *n* (%)	51 (38.1)	37 (37.4)	14 (40.0)		
12–14 points, *n* (%)	67 (50.0)	50 (50.5)	17 (48.6)		
BMI, kg/m^2^, M (SD)	29.6 (6.1)	29.2 (6.2)	30.8 (5.9)	0.19	
BMI ≥ 30 kg/m^2^, *n* (%)	62 (46.6)	43 (43.9)	19 (54.3)	0.33	
BMI < 18.5 kg/m^2^, *n* (%)	3 (2.3)	3 (3.1)	0 (0)	0.57	
WC, cm, M(SD)	95.2 (14.5)	92.0 (13.4)	105 (13.3)	<0.001	
Abdominal obesity, *n* (%)	102 (81)	77 (81.1)	25 (80.6)	1.00	8
Sarcopenia consensus parameters					
SARCF, points, Me (IQR)	2.0 (1, 5)	3 (1, 5)	2 (1, 3)	0.04	-
SARCF ≥ 4 points, *n* (%)	53 (39.6)	45 (45.5)	8 (22.9)	0.03	-
HGS, kg, Me (IQR)	20.3 (15.4, 25.3)	19.3 (14.2, 21.7)	30.1 (22.6, 35.2)	<0.001	-
Dynapenia, *n* (%)	50 (37.3)	35 (35.4)	15 (42.9)	0.28	-
Gait speed, m/s, Me (IQR)	0.73 (0.52, 0.93)	0.72 (0.52, 0.88)	0.79 (0.53, 1.16)	0.22	10
Slow gait (≤0.8 m/s), *n* (%)	76 (61.3)	58 (64.4)	18 (52.9)	0.39	10
TUG test, s, Me (IQR)	15.6 (11, 22.4)	16 (12, 23)	13.5 (9.5, 22.1)	0.10	7
TUG ≥ 20 s, *n* (%)	39 (30.7)	30 (32.6)	9 (25.7)	0.52	7
Biochemical parameters					
Serum protein, g/dL, M (SD)	6.5 (0.5)	6.5 (0.5)	6.6 (0.5)	0.95	
Serum protein < 6 g/dL, *n* (%)	18 (15.4)	12 (14.3)	6 (18.2)	0.58	
Serum albumin, g/dL, M (SD)	4.13 (0.38)	4.15 (0.37)	4.05 (0.41)	0.21	
Serum albumin < 3.5 g/dL, *n* (%)	7 (5.3)	5 (5.1)	2 (5.7)	1.0	
CRP ≥ 5 mg/L, *n* (%)	34 (25.4)	22 (22.2)	12 (34.3)	0.18	
IL-6, pg/mL, Me (IQR)	4.34 (2.79, 8.70)	3.81 (2.68, 8.68)	5.62 (3.54, 8.76)	0.14	
IL-6 > 8 pg/mL, *n* (%)	35 (26.1)	26 (26.3)	9 (25.7)	1.00	
TNF-α, pg/mL, Me (IQR)	25.73 (10.31, 61.57)	26.28 (10.52, 57.80)	18.86 (9.88, 101.86)	0.91	
TNF-α > 15 pg/mL, *n* (%)	81 (60.4)	61 (61.6)	20 (57.1)	0.69	
Hemoglobin, g/dL, M(SD)	12.5 (1.5)	12.3 (1.5)	13.1 (1.4)	0.01	
Anemia, *n* (%)	54 (40.3)	37 (37.4)	17 (48.6)	0.32	
Lymphocytes, K/µL, Me (IQR)	1.51 (1.22, 1.93)	1.59 (1.21, 1.93)	1.43 (1.26, 2.0)	0.36	
TSH, µIU/mL, Me (IQR)	1.36 (0.79, 2.01)	1.19 (0.79, 2.05)	1.52 (0.92, 2.0)	0.29	
NT-proBNP, pg/mL, Me (IQR)	357 (184, 784)	391 (191, 793)	303 (179, 518)	0.28	
GFR, mL/min/1.73 m^2^, Me (IQR)	60.9 (45.0, 82.6)	54,4 (43.9, 78.1)	70.3 (46.6, 97.8)	0.02	11
GFR < 60 mL/min/1.73 m^2^, *n* (%)	65 (48.5)	53 (53.5)	12 (34.3)	0.08	11
Serum creatinine, mg/dL, Me (IQR)	0.86 (0.71, 1.07)	0.79 (0.68, 1.03)	0.97 (0.86, 1.21)	<0.001	11
DHEA, ng/mL, Me (IQR)	0.44 (0.30, 0.57)	0.45 (0.30, 0.58)	0.43 (0.28, 0.56)	0.95	
25(OH)D, ng/mL, Me (IQR)	28.7 (18.2, 42.4)	32.8 (21.4, 43.3)	22.1 (13.1, 31.0)	0.002	2
Redox balance parameters					
TOS, μmol/L, Me (IQR)	656.5 (402.2, 1033.8)	729.0 (408.2, 1042.3)	555.6 (365.4, 986.4)	0.31	
TAS, μmol/L, Me (IQR)	358.4 (280.5, 385.8)	367.3 (294.2, 386.0)	336.4 (259.1, 379.6)	0.14	
OSI, M (SD)	2.24 (1.01)	2.27 (1.00)	2.15 (1.04)	0.46	

Where BMI—body mass index; CHF—chronic heart failure; CKD—chronic kidney disease; COPD—chronic obstructive pulmonary disease; CRP—C-reactive protein; DHEA—dehydroepiandrosterone; GDS—Geriatric Depression Scale; GFR—glomerular filtration rate; HGS—hand grip strength; IADL—instrumental activities of daily living; IHD—ischemic heart disease; IL-6—interleukin 6; IQR—interquartile range; M—mean; Me—median; MI—myocardial infarction; MNA-SF—Mini Nutritional Assessment-Short Form; n—number; OSI—oxidative stress index; PAD—peripheral arterial disease; SD—standard deviation; TAS—total antioxidative status; TNF-α—tumor necrosis factor α; TSH—thyroid-stimulating hormone; TOS—total oxidative status; TUG—Timed Up and Go test; WC—waist circumference; 25(OH)D—25-hydroxycholecalciferol. ^1^ Student’s *t*-test or the Mann–Whitney U test.

**Table 2:** Characteristics of people with dynapenia+ and without dynapenia−.

Characteristic	Dynapenia−	Dynapenia+	*p* ^1^
*n* (%)	84 (62.7)	50 (37.3)	
Sociodemographic characteristics			
Age (y), M (SD)	77.3 (7.3)	82.3 (6.3)	<0.001
Sex (women)	64 (76.2)	35 (70.0)	0.54
Chronic diseases			
Hypertension, *n* (%)	71 (84.5)	47 (94.0)	0.17
Atrial fibrillation, *n* (%)	14 (16.7)	13 (26.0)	0.27
CHF, *n* (%)	29 (34.5)	28 (56.0)	0.02
IHD, *n* (%)	13 (15.5)	12 (24.0)	0.26
MI, *n* (%)	1 (1.2)	4 (8.0)	0.06
PAD, *n* (%)	17 (20.2)	5 (10.0)	0.15
Brain infarct, *n* (%)	4 (4.8)	4 (8.0)	0.47
Orthostatic hypotension, *n* (%)	27 (32.1)	11 (22.0)	0.24
COPD, *n* (%)	3 (3.6)	3 (6.0)	0.67
Asthma, *n* (%)	1 (1.2)	0 (2.0)	1.00
Diabetes, *n* (%)	45 (53.6)	30 (60.0)	0.48
CKD, *n* (%)	26 (31.0)	21 (42.0)	0.26
Osteoarthritis, *n* (%)	5 (6.0)	6 (12.0)	0.33
Osteoporosis, *n* (%)	30 (35.7)	23 (46.0)	0.28
Neoplasm, *n* (%)	4 (4.8)	7 (14.0)	0.10
Dementia, *n* (%)	6 (7.1)	2 (4.0)	0.71
Depression, *n* (%)	56 (66.7)	34 (68.0)	1.00
Number of chronic diseases, Me (IQR)	4 (3.5)	5 (4.6)	0.005
Multimorbidity (2+ diseases), *n* (%)	77 (91.7)	50 (100.0)	0.045
Functional abilities and physical activity			
Barthel Index, points, Me (IQR)	95 (90, 100)	90 (70, 95)	0.03
ADL dependence, *n* (%)	6 (7.1)	16 (32.0)	<0.001
Duke OARS IADL, points, Me (IQR)	10 (8, 12)	8 (5, 10)	<0.001
IADL dependence, *n* (%)	29(34.5)	29 (58.0)	0.01
GDS, points, Me (IQR)	4 (3, 7)	5 (3, 7)	0.41
GDS 15 > 4 points, *n*(%)	40 (48.2)	29 (58.0)	0.29
Short-Blessed points, Me (IQR)	4 (2, 8)	6 (2, 12)	0.10
Short-Blessed > 9 points, *n* (%)	16 (19.3)	19 (38.0)	0.03
Low physical activity, *n* (%)	23 (28.0)	22 (44.0)	0.09
Nutritional and biochemical parameters			
MNA-SF, points, Me (IQR)	12 (9, 13)	11 (9, 13)	0.08
MNA-SF < 8, *n* (%)	9 (9.5)	8 (16.0)	0.09
8–11, *n* (%)	28 (33.3)	23 (46.0)	
12–14, n (%)	48(57.1)	19 (38.0)	
BMI, kg/m^2^, M (SD)	30.5 (6.0)	28.1 (6.2)	0.03
BMI > 30 kg/m^2^, *n* (%)	45 (53.6)	17 (34.0)	0.03
BMI < 18.5 kg/m^2^, *n* (%)	1 (1.2)	2 (4.0)	0.56
WC, cm, M (SD)	96.1 (15.0)	93.7 (13.6)	0.34
Abdominal obesity, *n* (%)	64 (84.2)	38 (76.0)	0.26
Sarcopenia consensus parameters			
SARCF, points, Me (IQR)	3 (1, 4)	4 (2,6)	<0.001
SARCF ≥ 4 points, *n* (%)	25 (29.8)	28 (56.0)	0.003
HGS, kg, Me (IQR)	22.0 (19.9, 29.4)	14 (9.8, 16.7)	<0.001
Men	34.3 (31.1, 37.9)	22.4 (18.3, 24.5)	<0.001
Women	21.1 (19.5, 24.4)	11.8 (7.9, 14.4)	<0.001
Gait speed, m/s, Me (IQR)	0.84 (0.64, 1.12)	0.54 (0.39, 0.72)	<0.001
Slow gait (≤0.8 m/s), *n* (%)	37 (46.3)	39 (88.6)	<0.001
TUG test, s, Me (IQR)	13 (9.4, 18)	20.9 (16.0, 32.5)	<0.001
TUG ≥ 20 s, *n*(%)	15 (18.5)	24 (52.2)	<0.001
Biochemical parameters			
Serum protein, g/dL, M (SD)	6.6 (0.43)	6.5 (0.68)	0.79
Serum protein < 6 g/dL, *n* (%)	7 (9.7)	11 (24.4)	0.04
CRP ≥ 5 mg/L, *n* (%)	20 (23.8)	14 (28.0)	0.68
Serum albumin, g/dL, M (SD)	4.19 (0.30)	4.02 (0.48)	0.03
Serum albumin < 3.5 g/dL, *n* (%)	2 (2.4)	5 (10.0)	0.10
Hemoglobin, g/dL, M(SD)	12.7 (1.5)	12.2 (1.5)	0.05
Lymphocytes, K/µL, Me (IQR)	1.53 (1.27, 1.96)	1.48 (1.09, 1.85)	0.37
TSH, µIU/mL, Me (IQR)	1.32 (0.87, 2.13)	1.40 (0.51, 1.83)	0.24
Serum creatinine, mg/dL, Me (IQR)	0.84 (0.70, 1.05)	0.88 (0.74, 1.21)	0.31
NT-proBNP, pg/mL, Me (IQR)	292 (171,509)	556 (304, 1773)	0.001
GFR, mL/min/1.73 m^2^, Me (IQR)	69.4 (46.6, 89.7)	53.7 (40.0, 69.9)	0.002
GFR < 60 mL/min/1.73 m^2^, *n* (%)	35 (41.7)	30 (60.0)	0.05
IL-6, pg/mL, Me (IQR),	4.34 (2.66, 7.36)	4.49 (2.87, 9.32)	0.43
IL-6 > 8 pg/mL, *n* (%)	19 (22.6)	16 (32.0)	0.31
TNF-α, pg/mL, Me (IQR)	26.52 (9.89, 61.78)	22.75 (11.36, 60.87)	0.77
TNF- > 15 pg/mL, *n* (%)	53 (63.1)	28 (56.0)	0.47
DHEA, ng/mL, Me (IQR)	0.46 (0.34, 0.62)	0.42 (0.20, 0.53)	0.03
25(OH)D, ng/mL, Me (IQR)	32.8 (18.2, 42.6)	26.5 (18.7, 39.8)	0.40

Where BMI—body mass index; CHF—chronic heart failure; CKD—chronic kidney disease; COPD—chronic obstructive pulmonary disease; CRP—C-reactive protein; DHEA—dehydroepiandrosterone; GDS—Geriatric Depression Scale; GFR—glomerular filtration rate; HGS—hand grip strength; IADL—instrumental activities of daily living; IHD—ischemic heart disease; IL-6—interleukin 6; IQR—interquartile range; M—mean; Me—median; MI—myocardial infarction; MNA-SF—Mini Nutritional Assessment-Short Form; n—number; OSI—oxidative stress index; PAD—peripheral arterial disease; SD—standard deviation; TAS—total antioxidative status; TNF-α—tumor necrosis factor α; TSH—thyroid-stimulating hormone; TOS—total oxidative status; TUG—Timed Up and Go test; WC—waist circumference; 25(OH)D—25-hydroxycholecalciferol. ^1^ Student’s *t*-test or the Mann–Whitney U test.

**Table 3:** Dynapenia, redox balance status, and sex.

Parameter	Dynapenia+	Dynapenia−	*p* ^1^
Total Group			
TOS, μmol/L, Me (IQR)	815.1 (398.4, 1189.8)	640.9 (403.9, 959.6)	0.12
TAS, μmol/L, Me (IQR)	346.3 (273.7, 381.7)	370.7 (287.4, 386.1)	0.25
OSI, M (SD)	2.52 (1.17)	2.06 (0.87)	0.02
Women			
TOS, μmol/L, Me (IQR)	819.3 (497.1, 1254.1)	653.21 (397.8, 963.2)	0.004
TAS, μmol/L, Me (IQR)	352.9 (298.5, 385.8)	376.6 (293.1, 386.1)	0.4
OSI, M (SD)	2.67 (1.08)	2.04 (0.90)	0.004
Men			
TOS, μmol/L, Me (IQR)	401.2 (335.5, 1168.3)	597.2 (463.4, 837.0)	0.73
TAS, μmol/L, Me (IQR)	336.4 (259.2, 378.5)	350.27 (255.0, 385.2)	0.66
OSI, M (SD)	2.17 (1.32)	2.13 (0.81)	0.92

Where IQR—interquartile range; M—mean; Me—median; SD—standard deviation; TOS—total oxidative status; TAS—total antioxidative status; OSI—oxidative stress index. “Dynapenia+”—hand grip strength <16 kg in women and <27 kg in men. ^1^ Student’s *t*-test or the Mann–Whitney U test.

**Table 4:** Determinants of dynapenia (low HGS) status—direct multivariable logistic regression models.

	OR	95% CI	*p*	OR	95% CI	*p*
		MODEL 1			MODEL 2	
Age, years	1.13	1.02–1.26	**0.03**	1.14	1.02–1.28	**0.02**
CHF	2.71	0.67–11.06	0.16	2.54	0.61–10.52	0.20
MI	19.01	1.20–302.24	**0.04**	18.42	1.03–329.80	0.05
Neoplasm	5.28	0.74–37.55	0.10	4.02	0.55–29.23	0.17
Number of chronic diseases	0.95	0.62–1.45	0.81	0.96	0.63–1.47	0.86
MNA-SF, points	0.92	0.71–1.18	0.50	0.92	0.71–1.18	0.49
BMI, kg/m^2^	0.90	0.80–1.01	0.06	0.90	0.80–1.01	0.06
Serum protein < 6 g/dL	0.59	0.09–3.85	0.58	0.56	0.08–3.66	0.54
Hemoglobin, g/dL	1.42	0.90–2.25	0.13	0.20	0.86–2.15	0.20
NT-proBNP, pg/mL	1.00	1.00–1.00	0.68	1.00	1.00–1.00	0.67
GFR, mL/min/1.73 m^2^	0.99	0.96–1.02	0.46	0.99	0.96–1.02	0.40
DHEA, ng/mL	0.09	0.01–1.01	0.05	0.08	0.01–0.88	**0.04**
OSI	1.87	1.05–3.34	**0.04**	1.91	1.06–3.46	**0.03**
Serum albumin (g/dL)	0.34	0.05–2.19	0.26	0.34	0.05–2.22	0.26
Low physical activity	0.87	0.28–2.73	0.81	0.92	0.29–2.95	0.89
GDS (points)	1.01	0.85–1.19	0.94	1.03	0.86–1.22	0.77
Short-Blessed test (points)	1.02	0.92–1.13	0.68	1.01	0.90–1.12	0.91
Sex (men)				1.98	0.51–7.60	0.32

Where BMI—body mass index; CHF—chronic heart failure; CI—confidence interval; DHEA—dehydroepiandrosterone; GDS—Geriatric Depression Scale; GFR—glomerular filtration rate; HGS—hand grip strength; MI—myocardial infarction; MNA-SF—Mini Nutritional Assessment-Short Form; OR—odds ratio; OSI—oxidative stress index; “Dynapenia+”—hand grip strength <16 kg in women and <27 kg in men. *p* value of less than 0.05 was regarded as significant. Bold signifies a statistically significant value.

**Table 5:** Determinants of low HGS status—stepwise backward multivariable logistic regression analysis.

	OR	95% CI	*p*
		MODEL 3	
Age (years)	1.14	1.05–1.24	**0.002**
BMI	0.89	0.82–0.98	**0.01**
OSI	1.78	1.08–2.95	**0.02**
DHEA	0.11	0.02–0.88	**0.04**
MI	10.81	0.95–123.56	0.06
Neoplasm	6.53	0.96–44.26	0.06
CHF	2.30	0.86–6.16	0.10

Variables included in the analysis: age, heart failure, history of myocardial infarction, neoplasm, number of chronic diseases, MNA-SF, BMI, serum protein < 6/dL, hemoglobin, NT-proBNP, GFR, DHEA, OSI, serum albumin, low physical activity, GDS, Short-Blessed test, and sex. *p* value of less than 0.05 was regarded as significant. Bold signifies a statistically significant value.

## Data Availability

The original contributions presented in this study are included in the article/Supplementary Materials. Further inquiries can be directed to the corresponding author.

## Supplementary Materials

The following supporting information can be downloaded at: https://www.mdpi.com/article/10.3390/nu17213413/s1, File S1: RawData.xlsx.

## Abbreviations

The following abbreviations are used in this manuscript:

ADL	Activities of daily living
AF	Atrial fibrillation
BMI	Body mass index
CHF	Chronic heart failure
CI	Confidence interval
CKD	Chronic kidney disease
CRP	C-reactive protein
CV	Coefficient of variation
COPD	Chronic obstructive pulmonary disease
DHEA	Dehydroepiandrosterone
DHEAS	Dehydroepiandrosterone sulfate
ELISA	Enzyme-linked immunosorbent assay
HGS	Hand grip strength
GDS	Geriatric Depression Scale
GFR	Glomerular filtration rate
Hb	Hemoglobin
EWGSOP2	The Second European Working Group on Sarcopenia in Older People
IADL	Instrumental activities of daily living
IHD	Ischemic heart disease
IL-6	Interleukin 6
IQR	Interquartile range
M	Mean
Me	Median
MI	Miocardial infarction
MNA-SF	Mini Nutritional Assessment-Short Form
NT-proBNP	N-terminal pro-B-type natriuretic peptide
N	Number
25(OH)D	25-hydroxycholecalciferol
OR	Odds ratio
OSI	Oxidative stress index
PAD	Peripheral arterial disease
ROS	Reactive oxygen species
RyR	Ryanodine receptor
SARC-F	S(trength), A(ssistance with walking), R(ise from a chair), C(limbing stairs), and F(alls) questionnaire
SCr	Serum creatinine
SD	Standard deviation
TAS	Total antioxidative status
TNF-α	Tumor necrosis factor α
TSH	Thyroid-stimulating hormone
TOS	Total oxidative status
TUG	Timed Up and Go test
WC	Waist circumference
